# Revisiting Bap Multidomain Protein: More Than Sticking Bacteria Together

**DOI:** 10.3389/fmicb.2020.613581

**Published:** 2020-12-23

**Authors:** Jaione Valle, Xianyang Fang, Iñigo Lasa

**Affiliations:** ^1^Instituto de Agrobiotecnología, CSIC-Gobierno de Navarra, Mutilva, Spain; ^2^Beijing Advanced Innovation Center for Structural Biology, School of Life Sciences, Tsinghua University, Beijing, China; ^3^Laboratory of Microbial Pathogenesis, Navarrabiomed-Universidad Pública de Navarra-Departamento de Salud, IDISNA, Pamplona, Spain

**Keywords:** Bap, biofilm, *Staphylococcus*, calcium, amyloid oligomers, adhesion

## Abstract

One of the major components of the staphylococcal biofilm is surface proteins that assemble as scaffold components of the biofilm matrix. Among the different surface proteins able to contribute to biofilm formation, this review is dedicated to the Biofilm Associated Protein (Bap). Bap is part of the accessory genome of *Staphylococcus aureus* but orthologs of Bap in other staphylococcal species belong to the core genome. When present, Bap promotes adhesion to abiotic surfaces and induces strong intercellular adhesion by self-assembling into amyloid like aggregates in response to the levels of calcium and the pH in the environment. During infection, Bap enhances the adhesion to epithelial cells where it binds directly to the host receptor Gp96 and inhibits the entry of the bacteria into the cells. To perform such diverse range of functions, Bap comprises several domains, and some of them include several motifs associated to distinct functions. Based on the knowledge accumulated with the Bap protein of *S. aureus*, this review aims to summarize the current knowledge of the structure and properties of each domain of Bap and their contribution to Bap functionality.

## Introduction

**Figure 1:**
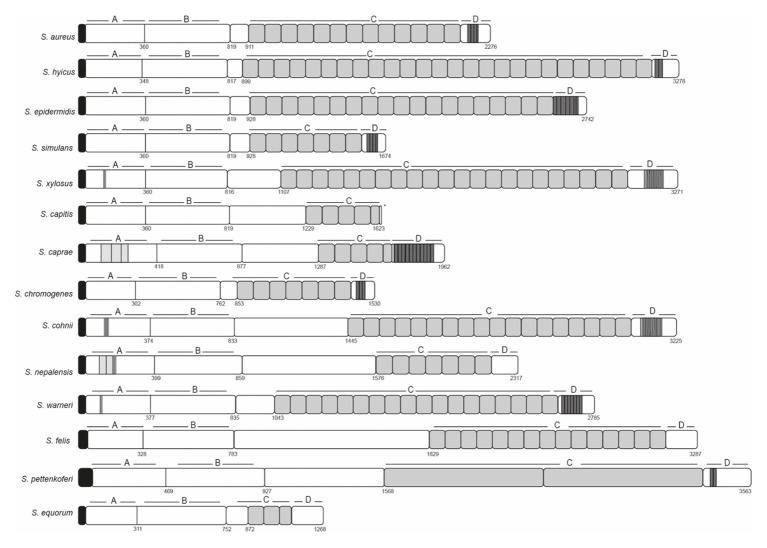
Schematic representation of staphylococcal Bap proteins. Structural organization in multidomains including the N-terminal domain (region A and B), core C-repeats domain and the carboxy-terminal domain (D). Repeats from A, C, and D domain are shown in gray. Signal peptide is shown in black.

Biofilm associated protein (Bap) was identified during the analysis of biofilm defective transposon insertion mutants in the bovine mastitis isolate, *Staphylococcus aureus* V329 (Cucarella et al., 2001). *S. aureus* V329 strain had been selected among a collection of bovine mastitis isolates for its strong capacity to produce biofilm in the classical microtiter plate assay. Sequencing of the *bap* gene surrounding region revealed that Bap was contained in a pathogenicity island (SaPIbov2; Ubeda et al., 2003). The finding showing the connection of Bap with the biofilm phenotype was somehow serendipitous. The integrase gene (*sip*) of SaPIbov2 in *S. aureus* V329 carried two-point mutations that abrogated the integrase functionality. As a consequence, SaPIbov2 remained stable in the bacterial chromosome. If the integrase would have been functional, most of the biofilm deficient mutants had been due to the loss of the SaPI element and not to genetic inactivation caused by transposon insertion. The presence of Bap in a pathogenicity island was anticipating that the fitness cost imposed by SaPIbov2 carriage was compensated by the benefit that the SaPI cargo (*bap* gene and the corresponding ABC transporter) conferred during the intramammary gland infection. Initial searches for Bap orthologs revealed the presence of Bap orthologs in many different bacterial species (Lasa and Penadés, 2006). The recent sequencing of hundreds of genomes from clinical and commensal isolates of different staphylococcal species has confirmed that *bap* gene is widely spread in the genomes of staphylococcal species (Tormo et al., 2005; Potter et al., 2009; Schiffer et al., 2019). Very often, the *bap* genes are carried in mobile genetic elements. However, there is no indication that the *bap* locus is carried on a mobile genetic element in some staphylococcal species such as *Staphylococcus epidermidis*, *Staphylococcus simulans*, *Staphylococcus chromogenes*, and *Staphylococcus warneri*. Bap is a large protein that comprise multiple domains, each of them representing an independent folding unit (Figure 1; Table 1). Since the discovery of the Bap protein, most studies have been directed to investigate how this protein induces adhesion and biofilm development. Surprisingly, it has been shown that a large part of the protein is not required for adhesion-accumulation biofilm phenotype and that Bap-mediated biofilm development occurs in response to environmental cues. However, there is still very little knowledge about the function of a large part of Bap protein and how different domains of Bap interact to facilitate Bap functionality. In this review, we update the current understanding of the structure and function of each region of Bap. Due to the absence of structural biology data, the regions have been defined arbitrarily based on the specific features of the primary amino acid sequence. Most of the available information has been obtained on the Bap protein of *S. aureus*, but it is assumed that conclusions drawn from studies with this protein can most likely be applied to other Bap orthologs.

**Table 1:** Staphylococcal Bap protein orthologs.

	Accession number	Length (amino acids)				Number repeats				Coiled-coil (position)	EF-hands (position)
		Protein	A-domain	B-domain	C-domain	A-repeat	C-repeat	SD-repeat	D-repeat		
*S. aureus*	AAK38834.2	2,276	45–360	361–819	911–2,116	–	14	3.4	–	224–281	727-740; 752-762
*S. hyicus*	AAY28520.1	3,278	45–348	349–807	899–3,136	–	26	2.1	–	212–269	715-728; 740-750
*S. epidermidis*	AAY28519.1	2,742	45–360	361–819	928–2,562	–	19	7	–	227–281	727-740; 752-762
*S. simulans*	AAY28518.1	1,674	45–360	361–819	928–1,530	–	7	3.4	–	227–281	727-740; 752-762
*S. xylosus*	AAY28517.1	3,271	45–360	361–816	1,107–2,998	2	22	–	17	67–110	723-736; 748-758
*S. capitis*	WP_080973601	1,623	45–360	361–819	1,229–1,609	–	4	–	–	227–281	727-740; 752-762
*S. caprae*	WP_170080878	1962	45–418	419–877	1,287–1,667	3	4	11.3	–	284–338	785-798; 810-820
*S. chromogenes*	AAY28516.1	1,530	45–302	303–761	853–1,370	–	6	3.6	–	169–223	669-682; 694-704
*S. cohnii*	WP_157947920	3,235	45–374	375–833	1,445–2,992	4	18	–	20	83–113	741-754; 766-776
*S. nepalensis*	WP_158266100	2,317	45–399	400–859	1,576–2,177	5	7	–	–	94–122, 150–191, 270–321	767-780; 792-802
*S. warneri*	WP_107536078	2,785	45–376	377–835	1,043–2,590	2	18	7	–	–	743-756; 768-778
*S. felis*	AVP36288.1	3,287	57–328	329–783	1829–3,118	–	15	–	–	–	–
*S. pettenkoferi*	ASE37144.1	3,563	76–469	470–927	1,568–3,304	–	2	–	2	353–380	836-840; 861-871
*S. equorum*	ALM58189	1,268	45–311	312–752	872–1,100	–	3	–	–	138–166	660-673; 685-695

## Multidomains of the Bap Protein

### N-Terminal Domain

#### Signal Peptide

**Figure 2:**
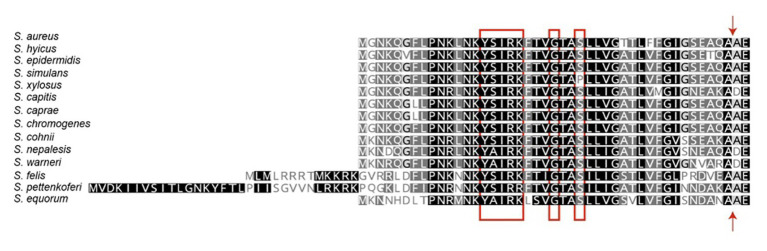
Signal peptides of staphylococcal biofilm associated proteins. The N-terminal signal peptides of Bap proteins were aligned at their predicted signal peptidase cleavage sites. The signal peptides harbor the YSIRK/GS motif (red box). The signal cleavage site is indicated by an arrow.

The staphylococcal Bap proteins are covalently linked to the cell wall envelope by a mechanism requiring the N-terminal secretory signal peptide and a C-terminal LPXTG motif sorting signal (Ton-That et al., 2000). Amino-acid sequences alignment of signal peptides from different staphylococcal Bap proteins revealed the presence of a consensus motif YSIRK/GS located 18–20 residues upstream of the signal peptide cleavage site (Figure 2; Dedent et al., 2007, 2008). This motif is conserved in the signal peptides of streptococci and staphylococci, but it is not present in the proteins of *Listeria monocytogenes*, bacilli, clostridia, or nocardia (Rosenstein and Gotz, 2000; Tettelin et al., 2001; Bae and Schneewind, 2003). In *S. aureus*, the YSIRK/GS is present in several cell-wall proteins such as clumping factor A (ClfA), Spa, fibronectin-binding protein B (FnbpB), serine-aspartate repeat protein C (SdrC) and D (SdrD; Dedent et al., 2008). Surface proteins transported through a signal peptide that contained the YSIRK/GS motif are deposited within the peptidoglycan cell wall at discrete foci and distributed in a ring-like or hemispherical manner perpendicular to the cell division plane. On the contrary, proteins secreted *via* signal peptides that lacked the YSIRK/GS motif are distributed in discrete, punctate deposits in the cell wall envelope (Dedent et al., 2007, 2008). Considering that Bap promotes adhesion to biotic and abiotic surfaces as well as intercellular adhesion, it seems reasonable that the protein needs to be evenly distributed on the cell surface to perform efficiently this function. We are not aware of any experimental evidence supporting that the distribution driven by the YSIRK/GS motif affects to Bap functionality, but this is an interesting hypothesis worthy of further study.

#### Region-A

**Figure 3:**
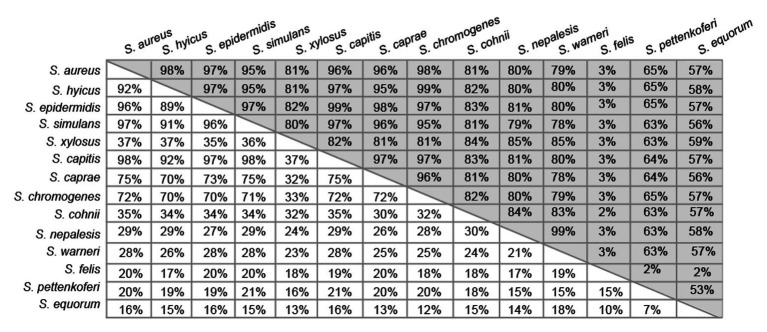
Pairwise percentage identity matrix between domains A (white boxes) and B (gray boxes) of Bap orthologs from the different staphylococcal species obtained from corresponding multiple sequence alignments done using *Geneious Prime* algorithm.

**Figure 4:**
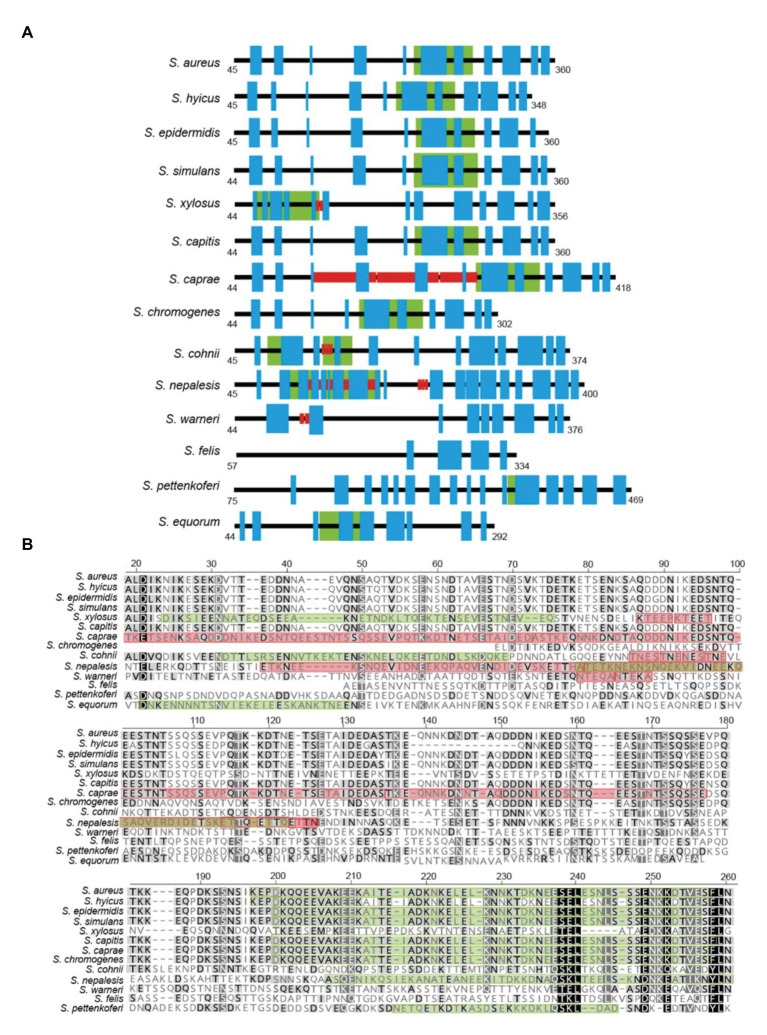
**(A)** Representative scheme of region A from staphylococcal Bap proteins. Blue boxes indicate the predicted alpha helices secondary structure. Red boxes indicate the presence of short repeats. Green boxes indicate the prediction of coiled-coil motifs. **(B)** Protein sequence alignment of a section of region A from staphylococcal Bap proteins. Alignments were generated using *Geneious Prime* multiple sequence alignment tool. Green box within each diagram indicate predicted coiled-coil domains. Red box within each diagram indicate amino acids repeats. Coiled-coil motif was predicted by J. M. Lupas program (https://npsa-prabi.ibcp.fr/cgi-bin/npsa_automat.pl?page=npsa_lupas.html). Repeats were detected using XSTREAM algorithm (https://amnewmanlab.stanford.edu/xstream).

Region A comprises ~250 to 400 amino acids (depending on the species) following the signal peptide (Figure 1). Sequence alignments of region A of Bap proteins from different staphylococcal species range from 98 to 10%. Overall, region A of Bap orthologs share an identity of 37%, being the highest conservation in the last amino acids of the domain. Domain A from *Staphylococcus capitis*, *S. simulans*, and *S. epidermidis* show sequence identity higher than 95% with domain A of Bap from *S. aureus* whereas domain A from *Staphylococcus equorum* shows the lowest sequence identity (10%; Figure 3). Region A can contain short repeats ranging from 4 to 56 amino acids, as it is the case for *Staphylococcus xylosus*, *Staphylococcus caprae*, *Staphylococcus cohnii*, *Staphylococcus nepalensis*, and *S. warneri* (Figures 4A,B). Secondary structure of region A is dominated by alpha helices (Figure 4A). Several informatic programs coincided to identify also coiled-coil motifs in Bap orthologs, except for the ones of *S. warneri* and *Staphylococcus felis*. Coiled-coil motifs are α-helical secondary structures mediating protein-protein interactions and multimerization through the coiling of helices that belongs to the same or different proteins (Parry et al., 2008; Fiumara et al., 2010). Intuitively, the presence of coiled coil secondary structure suggests that Bap-proteins could mediate intercellular adhesion through homophilic interactions between coiled-coil domains of opposing proteins in neighboring cells. Homophylic interactions between proteins of neighbor bacteria have been described for other staphylococcal surface proteins such as Aap and SasG. However, the homophylic interactions occurred between β-sheet–rich G5–E domains of Aap and SasG (Conrady et al., 2008, 2013; Geoghegan et al., 2010; Formosa-Dague et al., 2015). A *S. aureus* strain producing a derivative of Bap protein in which region A was deleted still maintained the capacity to form cell-to-cell aggregates and robust biofilms on polystyrene or on a glass surface under flow culture conditions, indicating that domain A of Bap is dispensable for these functions (Taglialegna et al., 2016b). Domain A could also be involved in the interaction with the host. Bap from *S. aureus* as well as Bap orthologs from *S. epidermidis*, *S. chromogenes*, and *Staphylococcus hyicus* are able to bind the host receptor Gp96 (Valle et al., 2012). The interaction of Bap with GP96 inhibits the entry of the bacteria into the cells by interfering with the fibronectin-binding protein mediated invasion pathway. Further studies are necessary to decipher whether domain A of Bap is involved in such interaction.

#### Region-B

**Figure 5:**
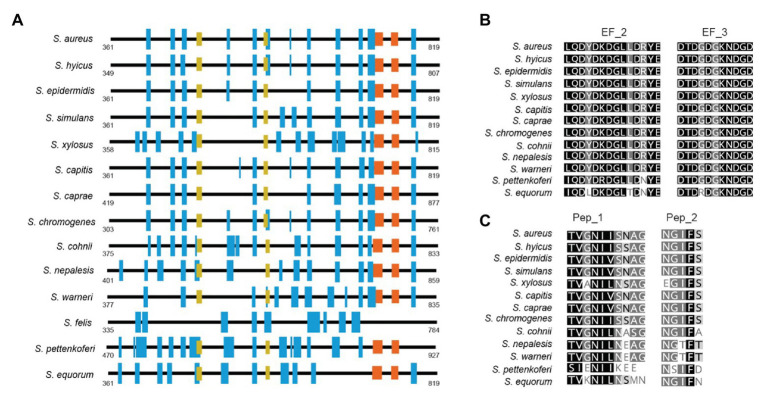
**(A)** Representative scheme of region B from staphylococcal Bap proteins. Blue boxes show the secondary structure of alpha helices. EF_Hand 2_3 motifs are represented by orange boxes. Yellow boxes show the localization of amyloidogenic peptides 1 (Pep_I) and 2 (Pep_II) present in Bap proteins. **(B)** Alignment between EF-Hand motifs from staphylococci Bap proteins. **(C)** Conservation of amyloidogenic peptides I and II present in Bap of *S. aureus* among staphylococcal Bap orthologs proteins. Conserved residues are shown in black. Secondary structures were predicted at GOR software (https://npsa-prabi.ibcp.fr/cgi-bin/npsa_automat.pl?page=/NPSA/npsa_gor4.html). Coiled-coil motif was predicted by J. M. Lupas program (https://npsa-prabi.ibcp.fr/cgi-bin/npsa_automat.pl?page=npsa_lupas.html). Repeats were detected using XSTREAM algorithm (https://amnewmanlab.stanford.edu/xstream).

Region B is comprised of the ~450 amino acids following region A (Figure 1). Region B is devoid of repetitions (Figure 5A). It is highly conserved among Bap orthologs (70% identity; Figure 3), and it exhibits similarity with Bap homologs from different bacterial genera, such as Esp protein from *Enterococcus faecalis* (Taglialegna et al., 2020). Region B of *S. aureus* contains two sequences with high similarity to the consensus EF-hand motif (amino acids 729–741 and amino acids 752–764). The EF-hand motif consists of a 12-residue loop flanked on both sides by a 12-residue alpha-helical domain and is involved in binding intracellular calcium (Strynadka and James, 1989). An early study aiming to determine the role of calcium in Bap functionality revealed that concentrations equivalent to those present in the milk (~10 mM) inhibit Bap-mediated bacterial aggregation and biofilm formation (Arrizubieta et al., 2004). This phenotype was dependent on the presence of EF-hand 2 and 3 because point mutations at positions 1, 3, and 12 of such motifs result in a Bap protein that still was able to induce biofilm development but it was unable to sense the presence of calcium in the media. These findings confirmed that EF-hand 2 and 3 constitute a basic stimulus-response coupling mechanism that allows Bap to induce or repress biofilm development depending on changes in the calcium concentration of the environment. With the exception of Bap from *S. felis*, all the staphylococcal Bap proteins present in the databases contain the conserved DxDxDG calcium-binding loop characteristic of the EF-hand motif, suggesting that calcium dependent regulation plays an important role in Bap functionality (Figure 5B). As we will see later, other regions of Bap might also be involved in the interaction with calcium.

Another interesting feature of region B is that it is self-sufficient to induce aggregation and biofilm phenotype. A *S. aureus* strain producing a chimeric protein containing the region B of Bap fused to ClfA protein conferred the same capacity to form biofilm than the entire Bap protein. Interestingly, the EF-hand motifs were still functional in the region B-ClfA chimeric protein because the biofilm induced by this protein was sensitive to the presence of calcium in the media and mutation of the EF-hand motifs renders the biofilm induced by the chimeric protein insensitive to the levels of calcium in the media (Taglialegna et al., 2016b). To add another layer of complexity to this region, a detailed analysis of region B sequence revealed the presence of two short stretches, peptide I _487_TVGNIISNAG_496_ and peptide II _579_GIFSYS_584_, with significant amyloidogenic potential (Taglialegna et al., 2016b; Figure 5C). These peptides as well as the purified recombinant Bap B region were able to form amyloid oligomers according to electron microscopy, attenuated total reflectance-Fourier transform infrared spectroscopy (ATR-FTIR), and binding to amyloid-related dyes such as Congo red, ThT, and Proteostat. Experimental evidence suggests that Bap is processed either enzymatically or non-enzymatically at the cell surface and fragments containing the N-terminal domain of the protein are released to the media. Later on, when the pH becomes acidic (pH < 5), the released region B of Bap undergoes a conformational change toward the amyloidogenic state resulting in the formation of fibrillar-like structures that mediate biofilm formation. The pH at which the region B of Bap shows aggregation activity is close to the isoelectric point of the region B peptide and therefore, the lack of net charge at this pH favors the conversion of the peptide from its native state (molten globule) to the amyloid-like state. At the same time, binding of calcium to the EF-hand domains of the protein stabilizes its molten-globule-like state, impairing their self-assembly into amyloid structures. Thus, the amyloid conformation of Bap is modulated by at least two environmental conditions, pH and the levels of calcium in the media.

This strategy to mediate multicellular behavior seems to be conserved among the Bap orthologs. Using the curli-dependent amyloid generator (C-DAG) system to detect amyloid aggregates (Sivanathan and Hochschild, 2013), it was shown that the region B from the Bap orthologs of *S. saprophyticus*, *Staphylococcus simiae*, *S. simulans*, *S. xylosus*, *S. epidermidis*, and region B from Esp of *E. faecalis* also displayed a similar amyloid-like properties (Taglialegna et al., 2020). Interestingly, amyloidogenic peptides similar to peptide I and peptide II were not present in the region B of Esp. However, this finding was not totally unexpected because the ability to form amyloid fibrils is a property inherent of the polypeptide chain, rather than a special property of any given sequence element. Other amyloidogenic peptides (sequence STVTVT) have been identified in C repeat region from *S. epidermidis*. Although the peptide is repeated 17 times in Bap protein of *S. epidermidis*, it is only present once in the Bap sequence of *S. aureus* (Lembre:2014jj). Thus, increasing number of evidence suggest that amyloid-like conformation is a general mechanism to mediate biofilm formation of the Bap_like proteins (Martino, 2016; Taglialegna et al., 2016a; Tursi and Tükel, 2018).

### Core-Repeats Region

In *S. aureus*, region C is comprised of a core of 14 tandem repeats of 86 amino acids (C-repeats), which are identical even at the nucleotide level (Cucarella et al., 2001). Although the function of this region has not been discerned, it has been proposed that it could have a structural role to maintain the proper protein conformation on the cell surface. The number of C-repeats varies significantly between the different staphylococcal species and even within the same bacterial species (Figure 1; Table 1; Tormo et al., 2005). Early reports demonstrated that variation in the number of C-repeats is a highly dynamic process because Bap proteins with different C-repeats were generated during the course of a staphylococcal infection most likely through homologous recombination events between identical repeats (Cucarella et al., 2004). Interestingly, the number of repeats had a negligible consequence on the protein functionality, because all the variants of Bap containing different number of C-repeats display similar capacity to induce biofilm development. A later study showed that a Bap protein derivative containing a single repetition (∆repBap) have similar capacity to mediate biofilm formation compared to the wild type strain and also, similar capacity to interact with the ligand GP96 protein, a protein expressed on the plasma membrane of different cell types (Valle et al., 2012). However, the interaction of the Bap derivative containing a single C-repeat with GP96 was less efficient inhibiting the entry of the bacteria into the cells probably because it causes lower steric impairment to the fibronectin-binding protein mediated invasion pathway (Dziewanowska et al., 1999; Joh et al., 1999; Fowler et al., 2000).

Homology search identified a Bap-like protein SiiE in *Salmonella*, which contains 53 tandem imperfect repeats that share significant similarities with the C-repeats of Bap from *S. aureus* (with an average of 25% identity; Latasa et al., 2005). These repeats are predicted to fold as a seven-strand β-sandwich and exhibit similarity to members of HYR (hyaline repeat) family that contain extracellular adhesion modules (Callebaut et al., 2000). Recently, the crystal structure of a SiiE fragment that covers repeats 50–52 of SiiE reveals the bacterial Ig-like (BIg) domain architecture and highlights two types of Ca^2+^-binding sites. Because the BIg domains of SiiE are highly homologous, the full-length adhesin SiiE is proposed to act as a calcium-rigidified, rod-like, surface-anchored molecule (Griessl et al., 2013). Given the significant similarities in C-repeats between SiiE and Bap, it is tempting to speculate that the structure and function of core C-repeats of Bap proteins are also regulated by Ca^2+^.

### Carboxy-Terminal Domain

The C-terminal domain of the *S. aureus* Bap protein (Region D) comprises three repeats of 18 amino acids and a cell-wall-anchoring region consisting of an LPXTG motif and hydrophobic amino acid segments characteristic of surface proteins covalently anchored to peptidoglycan. Region D repeats are present in many of the staphylococcal Bap proteins (Figure 1; Table 1). The repeats often contained sequence stretches rich in serine and aspartic acid residues resembling the SD repeats present in staphylococcal surface proteins as the serine-aspartate repeat proteins C, D, and E (SdrC, SdrD, and SdrE) and Clumping factor A (ClfA; Josefsson:1998bq). In other cases, the repeats included short amino acids stretches (3–5 amino acids) that are repeated 17–20 times.

The biological significance of repeats from region D and its implication for biofilm development remains poorly understood. SD repeats might acts as rigid rods to project the N-terminal domain further from the cell surface and certain number of SD repeats are required for functional expression of the ligand-binding domain on the cell surface (Hartford et al., 1997; Foster et al., 2014; Speziale et al., 2014). SD repeats are genetically unstable, and the variation in the number of SD repeats help bacteria to adapt with high efficiency and low cost to environmental changes (Cheng et al., 2012). The SD-repeat region of diverse surface proteins is often glycosylated (Hazenbos et al., 2013). Glycosylation protects protein from the proteolytic attack by human neutrophil. The implication of the SD repeats present in the carboxyl-terminal domain of Bap or the role of the post-translational modification of these repeats in the functionality of Bap awaits further studies.

## Role of Bap in Pathogenesis

The prevalence of *bap* in *S. aureus* and coagulase-negative staphylococcal isolates obtained from animal with mastitis is higher than in human isolates suggesting that specific host-dependent pathogenic factors involved in biofilm formation evolved independently in humans and ruminants (Cucarella et al., 2001; Tormo et al., 2005).

In lactating animals, the capacity to form biofilm on the epithelium of the mammary gland has been related to the propensity of staphylococci to produce chronic infections (Baselga et al., 1993, 1994; Cucarella et al., 2004). In this regard, the presence of Bap on the bacterial surface was shown to improve staphylococcal colonization and persistence on the mammary glands of infected animals (Cucarella et al., 2004).

Bap enhances the adhesion but inhibits the entry of *S. aureus* into epithelial cells (Cucarella et al., 2002, 2004). For that, Bap binds directly to the major chaperon Gp96 that is expressed on the surface of different cell types (Valle et al., 2012). The interaction of Bap with Gp96 may cause a steric impairment that hinders the recognition of FnBPs to fibronectin and integrins exposed on the cellular surface of the host cells, thus avoiding entry to host cells (Dziewanowska et al., 1999, 2000; Sinha and Fraunholz, 2010). Contrary to what happens with invasion, increased adhesion of Bap-positive strains was independent of the presence of Gp96 suggesting that Bap interacts with another factor to promote bacterial adhesion to epithelial cells.

In conclusion, Bap promotes adhesion to the epithelial cells of the mammary gland and impair bacterial internalization through the interaction with Gp96. This interaction facilitate the establishment of long-term persistent chronic infections by at least two mechanisms, generation of a microenvironment that acts as a physical barrier for host immune cells and shielding bacterial cells from detection by the immune system (Scherr et al., 2014; Schilcher and Horswill, 2020).

## Final Remarks

Future directions of research in Bap protein should considered at least two issues/questions. First, it would be very helpful to obtain a full-length structural model of Bap to understand how the interaction between the different domains or the function of putative active sites hidden at the interface between domains affects Bap biological functions. A structural model of Bap protein will aid to explain the phenotype of mutants generated in previous studies and more importantly in the designing of new mutants to disrupt or alter specific functions. Bap exemplifies the difficulties that exist with large multidomain proteins to close the gap between the increased information about the structure and function of single-domains and the requirement of high-resolution protein structures to understand protein functionality. To solve this problem, several multidomain structure modeling strategies have been recently developed (Xu et al., 2015; Georg et al., 2019; Zhou et al., 2019; Hou et al., 2020). The application of these algorithms for protein structure modeling is particularly challenging in the case of Bap because a global template that covers all individual domains is not available.

A second intriguing question is related with the presence of Bap in bovine mastitis isolates of *S. aureus* and their absence from human clinical isolates. Bap-mediated biofilm seems to be a system specialized for the conditions present in the mammary gland, where calcium concentration can reach the high values necessary to modulate Bap function (~10 mM). For calcium to serve as a regulator of Bap function, the fluctuations in the local calcium concentration should be higher than the binding affinity of the protein for the cation. Otherwise, Bap-mediated biofilm development will be irreversible, and bacterial propagation from the site of infection will be seriously compromised. It is very likely that only in the mammary gland environment the concentration reaches the high values needed to inhibit the function of Bap. It will be important to determine whether Bap-mediated biofilm is a system specialized for the conditions present in the mammary gland. If so, it will represent an excellent model to explore the real contribution of adhesion and biofilm development during the course of the infection process.

## Author Contributions

IL and JV conceptualized the content of the article. IL, JV, and XF contributed to writing and revision of the work. All authors contributed to the article and approved the submitted version.

### Conflict of Interest

The authors declare that the research was conducted in the absence of any commercial or financial relationships that could be construed as a potential conflict of interest.
